# Remdesivir may exacerbate ischemic acute kidney injury through molecular alterations in PGC-1α and apoptosis pathways: An *in vivo* study

**DOI:** 10.1371/journal.pone.0336221

**Published:** 2026-02-12

**Authors:** Yasin Bagheri, Zahra Malekinejad, Seyyedeh Mina Hejazian, Abdollah Abdollahpour, Fatemeh Khajepour, Mohaddeseh Farahbod, Sama Ahmadi, Samira Matin, Sepideh Zununi Vahed

**Affiliations:** 1 Kidney Research Center, Tabriz University of Medical Sciences, Tabriz, Iran; 2 Department of Pathobiology, Faculty of Veterinary Medicine, Tabriz Branch, Islamic Azad University, Tabriz, Iran; 3 Faculty of Veterinary Medicine, Tabriz Branch, Islamic Azad University, Tabriz, Iran; 4 Department of Clinical Science, Faculty of Veterinary Medicine, Tabriz Branch, Islamic Azad University, Tabriz, Iran; 5 Faculty of Medicine, Tabriz University of Medical Sciences, Tabriz, Iran; University of Coimbra: Universidade de Coimbra, PORTUGAL

## Abstract

Acute kidney injury (AKI) represents a significant complication in patients with COVID-19. Although Remdesivir (RDV) has been shown to reduce viral loads and improve clinical outcomes, concerns persist regarding its safety in individuals with pre-existing kidney impairment. This study investigated the effects of RDV on a rat model of ischemia/reperfusion (I/R)-induced kidney damage. A total of 24 rats were divided randomly into four groups: (1) control, (2) I/R, (3) I/R + RDV by intraperitoneal (ip) injections, and (4) I/R + RDV by subcutaneous (sc) injection groups. Rats in groups 3 and 4 received a single dosage of RDV (25 mg/kg) one hour before I/R induction. The effect of RDV on master genes involved in the mitochondrial biogenesis [Peroxisome proliferator-activated receptor gamma coactivator (PGC-1α)] and dynamics [Dynamin-related protein 1 (Drp-1)], cellular stress [Activating transcription factor 3 (ATF3)], inflammation [Nuclear factor kappa B (NF-κB)], cell death [p53, p21 (a cyclin-dependent kinase inhibitor), and caspase-3], as well as oxidant malondialdehyde (MDA) and antioxidant factors were evaluated. Moreover, renal function, along with histology assessments were studied. Significant reductions in mitochondrial biogenesis marker PGC-1α (*P* ≤ 0.04) and increases in caspase-3 (*P* = 0.003) expression levels were observed in the I/R + RDV + sc group compared to the I/R group. Oxidative stress marker was elevated (*P* = 0.016), while glutathione peroxidase (GPX) activity and total antioxidant capacity (TAC) were significantly decreased in the I/R + RDV + sc group (0.003 and 0.045, respectively). However, no significant changes were observed in p-p53, p-p21, NF-κB, or Drp-1 levels. Subcutaneous injection of RDV could induce more injury to the kidney compared to the intraperitoneal injection. These findings suggest that RDV may exacerbate AKI by hindering mitochondrial biogenesis and promoting renal cell apoptosis, without significantly affecting overall kidney function or histopathology. Clinically, these results highlight the need for caution when using RDV in patients with impaired renal function, especially during COVID-19 treatment.

## 1. Introduction

Acute kidney injury (AKI) is a sudden loss of kidney function due to a rapid decline in the glomerular filtration rate (GFR). Several pathological processes, including severe inflammatory and immunological processes, oxidative stress, and epithelial/endothelial cell death, contribute to AKI [1–3]. AKI occurs frequently during renal transplantation [4], major vascular surgery [5], sepsis [6], and among patients with coronavirus disease (COVID-19).

Remdesivir (RDV or GS-5734), as an antiviral nucleotide prodrug, inhibits viral loads and ameliorates disease outcomes in COVID-19 patients [7,8]. Infiltrated macrophages in the injured kidney that are polarized to pro-inflammatory type (M1) may be inhibited by RDV [9,10]. Based on clinical studies, RDV can improve kidney function, and there was no significant association between RDV and renal dysfunction in patients without a previous chronic kidney disease (CKD) [11].

Evidence has also shown a significant correlation between AKI incidence and RDV usage in COVID-19 patients with kidney impairment [12–14]. A higher proportion of these patients with severe renal impairment developed AKI [15]. Recently, it has been indicated that RDV has more adverse effects on AKI patients who are men and older than 65 years [14]. Moreover, it is reported that there is an increased risk of AKI in patients treated with RDV and recommended that RDV should not be used in patients with GFR less than 30 mL/min [12]. However, some other studies reported that RDV administration in COVID-19 patients with an estimated creatinine clearance (eCrCl) of <30 ml/min [16,17] and estimated GFR between 15 − 60 mL/min/1.73m^2^ [18] was not significantly associated with an increased risk of adverse kidney outcomes. No nephrotoxic effects of RDV was reported in patients with kidney dysfunction [19,20] and no association was found between AKI and RDV in different studies [12–14]. Similarly, the results of a systematic review revealed that RDV administration in patients with severe kidney injuries has not only low nephrotoxicity but also reduces COVID-19-induced AKI [21]. However, the safety data on RDV’s effect on renal function were too limited.

There is considerable controversy regarding the protective versus nephrotoxic effects of RDV on kidneys with impaired function. The nephrotoxic effects of RDV primarily stem from mechanisms involving mitochondrial dysfunction, such as the inhibition of mitochondrial RNA polymerase and the impairment of cellular energy metabolism, leading to subsequent cell death pathways [22]. Therefore, this study aimed to investigate the effects of RDV on ischemia/reperfusion (I/R)-induced kidney injury in a rat model and to explore its potential molecular mechanisms. So, the effect of RDV on kidney function and master genes involved in the biogenesis (PGC-1α) and dynamics (Drp-1) of mitochondria was studied at the protein levels. Moreover, master regulatory factors involved in cellular stress)ATF3: stress-responsive transcription factor(, inflammation (NF-κB), cell cycle regulation)p53 and p21(, apoptosis)caspase-3(, along with the oxidant/antioxidant factors [malondialdehyde (MDA), glutathione peroxidase (GPX), superoxide dismutase (SOD), and total antioxidant capacity (TAC)] were evaluated in kidney tissues.

## 2. Methods

In this experimental study, 24 adult male Wistar rats (220 ± 30 g weight) were purchased from Pasture Institute (Tehran, Iran). The sample size was obtained based on the rule of thumb for One-Way-ANOVA design animal studies [23]. For ten days, animals were kept in standard condition (12/12 hours of light/dark cycle and 22 °C) according to Animals Care and Use standards. Rats were divided randomly into 4 groups: [1] control group, [2] I/R group, [3] I/R group+ RDV by intraperitoneal (ip) injections, and [4] I/R + RDV by subcutaneous (sc) injection [10,24,25]. RDV was injected at a single dosage of 25 mg/kg [10,24,25] just one hour before kidney I/R.

Kidney I/R model was constructed as described previously [26,27]. For ischemia induction in groups 2–4, rats were anesthetized by injection of xylazine (10 mg/kg) and ketamine (90 mg/kg). Left renal arteries were blocked for 45 minutes by non-traumatic vascular clamps. The ischemic kidney was accepted when a pale-colored kidney was seen. Then, by removing the forceps, kidney reperfusion was established. In the sham group, only vascular manipulation in the left kidney was executed without the application of clamping. Then, to recover from anesthesia, the abdominal space of the animals was stitched up, and they were returned to their cages. Six hours after reperfusion, rat blood samples were collected, and then they were euthanized by ip injection of 200 mg/kg Thiopental sodium. The kidney of rats was separated and kept for further studies in liquid nitrogen. The study was permitted by the Ethics Committee of Tabriz University of Medical Sciences, Tabriz, Iran (Ethical code: IR.TBZMED.AEC.1401.083).

To assess the impact of RDV on the histopathological alterations following post-I/R injury, kidney tissue specimens were preserved in a 10% formaldehyde solution, subjected to a series of dehydration processes using alcohol, clarified with toluene, and subsequently embedded in paraffin wax. Thereafter, the specimens were stained utilizing hematoxylin and eosin (H&E) and examined under light microscopy (Olympus, Tokyo, Japan) [28] by a trained histologist who remained uninformed about the specifics of the study design. According to a 5-score system [29], infiltration of immune cells, the number of apoptotic/necrotic tubular cells, dilation of tubules, alterations in brush border, and formation of tubule cast were evaluated.

To assess the protein expression levels of mitochondrial, apoptotic, and inflammatory factors and ATF3 in the kidneys, the western blotting method was employed. Briefly, kidney tissues were stored in cold serum, immediately washed in very cold saline, and homogenized in cold RIPA lysis buffer containing a protease inhibitor (1%). Subsequently, they were centrifuged for 10 min at ×12,000 rpm and the total protein quantity in the supernatants was determined (Pierce Biochemical, Rockford). Sodium dodecyl-sulfate polyacrylamide gel electrophoresis (SDS-PAGE) (10%) was used for protein separation of each sample. The separated protein on the gel was then transferred to polyvinylidene fluoride and probed with primary antibodies. Monoclonal antibodies were against PGC-1α (ab54481, Abcam), NF-κB p65 (ab16502, Abcam), Drp-1 (sc-271583), ATF3 (sc-518032), p-p53 (sc-377553), p-p21 (sc-377569), and caspase-3 (sc-7272) (Santa Cruz Biotechnology, Inc). To normalize the signal intensity of each band, β-actin was used (sc-47778, Santa Cruz Biotechnology Inc.).

Serum urea and creatinine levels were also measured by colorimetric assay kits (BioMerieus). To assess the impact of RDV on kidney oxidative stress and antioxidant molecules, respectively, the levels of MDA and TAC were assessed using Elabscience (Catalog No: E-BC-K025-S, USA) and Randox (United Kingdom). Moreover, the activity of antioxidant enzymes, including GPX and SOD, was evaluated by Ransel and Ransod kits (Randox, United Kingdom), respectively, with a Multiscan GO microplate reader (Thermo Scientific, USA).

### 2.1. Statistical analysis

The Shapiro-Wilk test was used to check the normality of the data. The mean ± standard deviation was used to present the data. An analysis of variance (one-way ANOVA) test was conducted to compare the significance of differences between the studied groups, followed by multiple comparisons with Tukey’s post-hoc. Since the data of tissue injury score were ordinal, the results presented as Median (min-max) and compared using nonparametric Kruskal–Wallis test. The level of significance was defined as *P* < 0.05. Data analysis was performed using GraphPad Prism version 6.01 software.

## 3. Results

**Fig 1:**
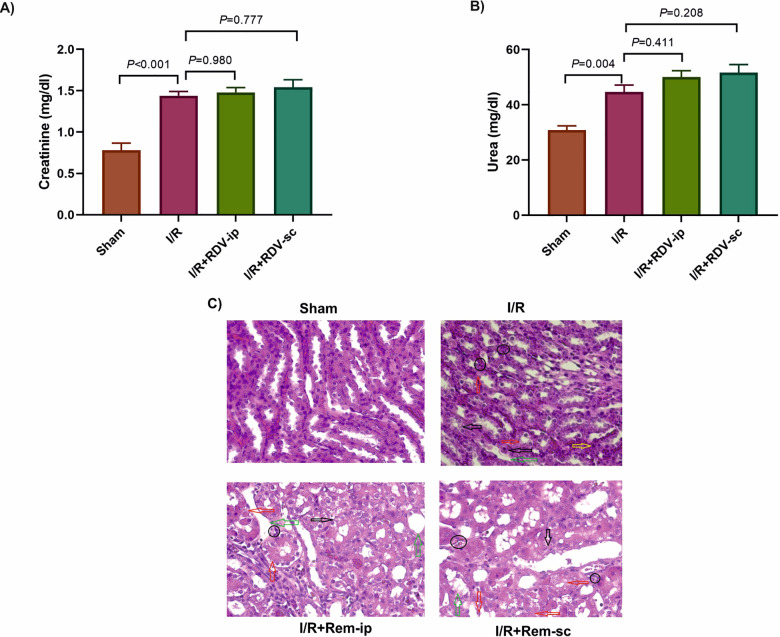
The effects of RDV on the kidney function and histology. The serum levels of creatinine **(A)** and urea **(B)** were compared between groups. Histological results of RDV were shown in the kidney tissue of the groups: O: Infiltration of inflammatory cells, Black arrows: Cellular degeneration and welling, Yellow arrows: Pyknosis of the nucleus, Red arrows: Necrosis of some kidney tissue cells, Green arrows: widening of the capsular space **(C)**. ip: intraperitoneal, I/R: ischemia/reperfusion, Rem: Remdesivir, sc: subcutaneous.

The protective or nephrotoxic effect of RDV was evaluated on I/R-induced kidney injury in rats. Serum creatinine (P < 0.001) and urea (*P* = 0.004) were increased significantly in the I/R group compared to the controls. No significant changes were seen in the serum creatinine levels between the I/R and the I/R + RDV + ip (*P* = 0.980) and I/R + RDV + sc (*P* = 0.777) groups (Fig 1A). Urea levels were not significantly different between the I/R + RDV + ip (*P* = 0.411) and I/R + RDV + sc (*P* = 0.208) groups in comparison to the I/R group (Fig 1B). The histological alterations in the studied groups are presented in Fig 1C. Infiltration of inflammatory cells, cellular degeneration and swelling, pyknosis of the nucleus, some kidney cell necrosis, and widening of the capsular space were observed in the I/R group. Accordingly, pyknosis of the nucleus disappeared in both I/R + RDV + ip and I/R + RDV + sc groups. Kidney damage score was increased in the I/R group compared to the sham group (Median of 3 [2–4], *P* < 0.001), and the non-significant damaging effects of RDV were observed in the kidney of I/R + RDV + ip and I/R + RDV + sc rats compared to the I/R group (*P* = 0.930).

**Fig 2:**
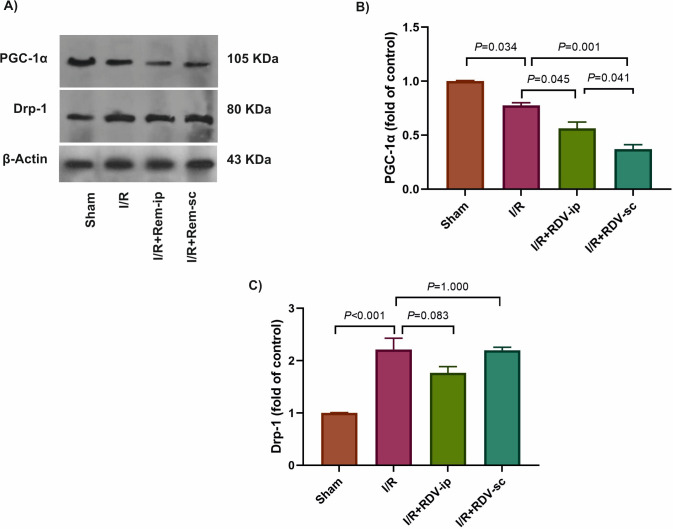
The effects of RDV on mitochondrial biogenesis and dynamics in the studied groups. The results of western blotting **(A)**. The protein levels of PGC-1α (B) and Drp-1 (C) were compared between the studied groups. β-actin was used as an internal control. Drp-1: Dynamin-related protein 1, ip: intraperitoneal, I/R: ischemia/reperfusion, PGC-1α: peroxisome proliferator-activated receptor-gamma coactivator, Rem: Remdesivir, sc: subcutaneous.

Mitochondrial dynamics is a quality control process of mitochondria that is preserved by a balance between fission and fusion, promoting mitochondrial fragmentation and elongation, respectively. To evaluate the impact of RDV on mitochondrial biogenesis and dynamics, the protein levels of PGC-1α and Drp-1 were evaluated in the kidneys of the studied rats (Fig 2A-C). Compared to the sham group, a significant reduction in the PGC-1α level and an elevation in the Drp-1 level were observed in the I/R group (*P* = 0.034 and *P* < 0.001, respectively), Fig 2B, 2C. RDV pre-treatment decreased the PGC-1α levels in the I/R + RDV + ip compared to the I/R group (*P* = 0.045), the decreased level was even more in the I/R + RDV + sc group (*P* = 0.001). Protein level of PGC-1α was less in the I/R + RDV + sc group than in the I/R + RDV + ip group (*P* = 0.041, Fig 2B). The increased levels of Drp-1 were similar between the I/R and RDV-treated I/R + RDV + sc and I/R + RDV + ip groups *P* ≥ 0.083 (Fig 2C). These results implied that RDV worsens the mitochondrial dysfunction in I/R-induced kidney injury.

**Fig 3:**
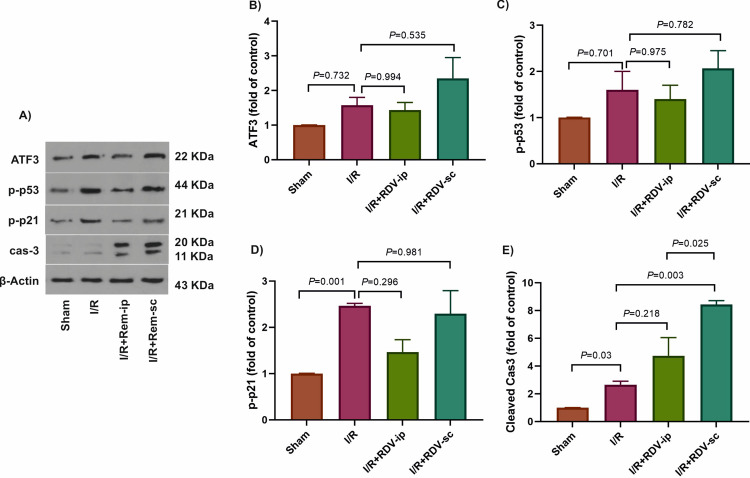
The effects of RDV on cellular stress in the studied groups. The results of western blotting **(A)**. The protein levels of ATF3 **(B)**, p-p53 **(C)**, p-p21 **(D)**, and cas3 **(E)** were represented in the studied groups. β-actin was used as an internal control. cas3: caspase-3, ip: intraperitoneal, I/R: ischemia/reperfusion, NF-κB: nuclear factor kappa-light-chain-enhancer of activated B cells, Rem: Remdesivir, sc: subcutaneous.

We also investigated if RDV disturbs the expression of the master ATF3, p-p53, and p-p21, and caspase-3 proteins involved in cellular stress, cell cycle regulation, and apoptosis (Fig 3A-E). ATF3 levels did not differ significantly in the I/R (*P* = 0.73) and RDV-treated rats (*P* ≥ 0.53), compared to the sham and I/R groups, respectively (Fig 3B). Since ATF3 overexpression inhibits oxidative stress-induced cell death [30,31], we hypothesized that increased ATF3 in the RDV-treated groups could preserve kidney cells against I/R-induced cell death. In so doing, the levels of p-53, p-21, and caspase 3 were also evaluated at the protein levels. In the I/R group, no statistically significant changes in p-p53 levels (*P* = 0.70, Fig 3C) and significantly high levels of p-p21 (*P* = 0.001, Fig 3D) were detected compared to the sham group. Notably, RDV had no significant effect on the p-p53 and p-p21 levels compared to the I/R group (*P* ≥ 0.29, Fig 3C, 3D). At the protein level, a significant upregulation of caspase-3 was observed in the I/R group compared to the sham group (*P* = 0.03). Likewise, a 4-fold increase was seen in caspase-3 level in the I/R + RDV + sc group compared to the I/R group (*P* = 0.003); however, in the I/R + RDV + ip group no significant difference was observed compared to the I/R group (*P* = 0.218), indicating that RDV pretreatment could worsen the levels of this apoptotic protein significantly (Fig 3E). Higher level of caspase-3 was more significant in the I/R + RDV + sc than the I/R + RDV + ip group (*P* = 0.025).

**Fig 4:**
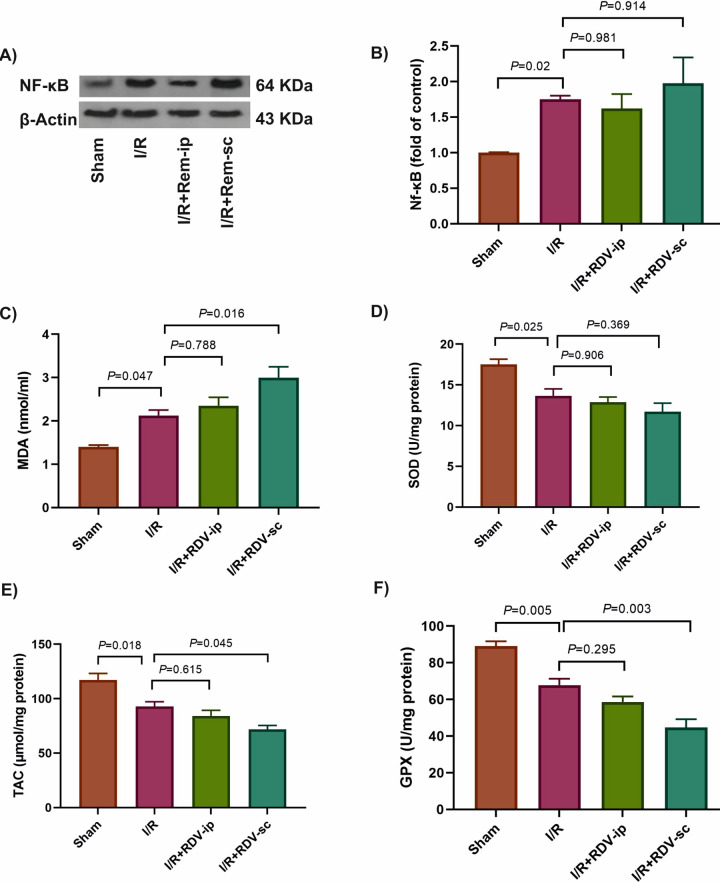
The effects of RDV on inflammatory and oxidative factors in the studied groups. The protein levels of NF-κB as an inflammatory marker were determined by western blotting and β-actin was used as an internal control **(A, B)**. The levels of oxidative markers including MDA **(C)** as an oxidative factor and SOD **(D)**, TAC **(E)** and GPX **(F)**, as antioxidative factors were evaluated in the studied groups. GPX: glutathione peroxidase, ip: intraperitoneal, I/R: ischemia/reperfusion, MDA: malondialdehyde, Rem: Remdesivir, sc: subcutaneous, SOD: superoxide dismutase, TAC: total antioxidant capacity.

The protein level of master inflammatory NF-κB was increased in the I/R group compared to the sham group (*P* = 0.02). However, RDV pre-treatment did not result in a decrease in the levels of NF-κB compared to the I/R group, *P* ≥ 0.91 (Fig 4A-B, Information S1 Raw Images).

In terms of the oxidative and antioxidative status of the studied kidneys, a remarkable increase in MDA levels (*P* = 0.04, Fig 4C) and a considerable reduction in SOD enzymatic activities (*P* = 0.02, Fig 4D) and TAC levels (*P* = 0.01, Fig 4E) were observed in the I/R groups compared with the sham group. No significant change was observed in SOD levels in the RDV-treated rats compared to the I/R group (P ≥ 0.369, Fig 4D). The enzymatic activity of GPX was significantly reduced in the I/R group compared to the sham group (*P* = 0.005, Fig 4F). A statistically significant elevation was observed in kidney MDA levels of RDV-treated groups compared to I/R rats, and this increase was even greater in the I/R + RDV + sc group (*P* = 0.016, Fig 4C). Moreover, a significant decrease in TAC levels and in the activity of GPX was observed in the kidneys of RDV-sc-treated rats compared to the I/R group (*P* = 0.045 and *P* = 0.003, respectively), and they were not significant in the I/R + RDV + ip groups (*P* = 0.615 *P* = 0.295 and, respectively, Fig 4E, 4F, S1 Raw Data).

## 4. Discussion

Remdesivir is an effective nucleotide analog drug against COVID-19, but its potential to cause or prevent I/R-induced AKI remains vague. The results of this study demonstrated that RDV may affect kidney cells by inducing mitochondrial dysfunction and promoting apoptosis.

Pharmacokinetic data have indicated that RDV and its active metabolites are mainly eliminated by kidneys (74%), hence, the drug’s potential toxicity concerns patients with kidney impairment [32,33]. RDV can induce mitochondrial injury and cellular toxicity that could be aggravated by hypoxia, occurring frequently in patients with severe COVID-19 [22].

The result of the present study indicated that RDV did not cause significant histological alterations in kidney tissues. A possible explanation for this finding is that the I/R model itself induces kidney damage, which may overshadow or mask any additional nephrotoxic effects of RDV. While specific animal studies on kidney histological effects of RDV are limited, the observed nephrotoxic effects in humans imply possible histological alterations, such as glomerular changes or tubular damage [12] that is compatible with our study. Moreover, in a study by Danaiyan et al, it is indicated that administration of RDV (17 mg/kg on the first day and 8.5 mg/kg for 9 days) in 12-week-old Wistar rats increased creatinine and urea levels compared to the control group [34] that was in contrast with the results of the present study.

A statistically significant decrease was observed in the PGC-1α levels in the I/R + RDV + ip and I/R + RDV + sc groups compared to the I/R group. Although Drp-1 (a master regulator of mitochondrial fission and fragmentation) showed a significant increase in the I/R group, in rats pretreated with RDV, no noticeable change was observed compared to the I/R group. These results showed that RDV induces renotoxicity via the downregulation of PGC-1α and decreased mitochondrial biogenesis. Moreover, up-regulation of the Drp-1 protein and consequently extreme mitochondrial fission and fragmentation participate in the pathogenesis of RDV-induced kidney injury. Remdesivir prevents human mitochondrial RNA polymerase weakly [35,36], inhibiting nuclear transcription of mitochondrial genes. Remdesivir has also several off-target impacts that may modify mitochondrial DNA homeostasis by the interruption of mitochondrial polymerases. Studies suggest tissue- and cell-specific effects of RDV [37]. In the brain, little or no effect of this drug was seen on mitochondrial respiration [38] while in the liver, intestinal cell lines (at high doses of 10 or 20 μM) [39], and cardiomyocytes [40] mitochondrial toxicity were observed.

At molecular levels, when organs are exposed to multiple stresses, including oxidative and mitochondrial stresses, ATF is induced to maintain cellular homeostasis [41,42]. An increased level of ATF3 was observed in the I/R-induced group and it was even higher in the I/R + RDV + sc group, implying a response of elevated stress and induction of I/R injury in the kidney cells in RDV-treated rats. Although this alteration was not statistically significant, the trend is consistent with other studies reporting that ATF3 can protect against I/R-induced inflammation [43,44], oxidation [45], apoptosis, or organ injury and remains weak under normal conditions. Furthermore, ATF3protects tubular cells against H_2_O_2_-induced I/R injury and cell death [31]. It should be noted that the expression of these factors can be time-dependent after ischemic periods and insignificant results may be influenced by other confounding conditions [46].

It has been reported that the protective effect of ATF3 against kidney I/R injury may be attributed to its regulation of p53 and p21, both of which are crucial proteins involved in cell cycle regulation. p53 regulates the DNA repair, cell cycle, and apoptosis. Its expression is induced by I/R, highlighting its pathogenic role in tubular injury and apoptosis associated with kidney I/R [47]. Conversely, the increased p21 may contribute to protection against oxidative stress by promoting cell-cycle arrest and inhibiting apoptosis [48]. In the present study, statistically significant increase was observed in p53 and p-p21 levels of I/R rats compared to the shams. Moreover, insignificant changes were observed in protein levels of p53 and p-p21 in the I/R + RDV + ip and I/R + RDV + sc groups compared with the I/R group. In agree with these results, previous studies have shown that p21 is rapidly and continuously upregulated in various models of AKI to protect renal tissues [49]. It is transcriptionally regulated by p53 to induce cell cycle arrest at the G1 phase, facilitating subsequent tubular cell proliferation after I/R [50]. ATF3 has been shown to inhibit cell death in umbilical vein endothelial cells and cardiac myocytes by downregulating p53 [30,51]. Therefore, the downregulation of p53 alongside the upregulation of p21 may play a protective role against kidney I/R injury. These findings align with our results. Furthermore, the expression levels of these factors may vary depending on the timing following ischemic events [46]. Given that tubular cell death through apoptosis or necrosis is a hallmark of kidney ischemic damage, caspase-3 level, which represents one of the final steps in apoptosis [52], was assessed in the present study. An increase was observed in the levels of this protein in RDV-treated groups, and it was significant in the RDV + I/R + sc compared to the I/R group. These results suggest that ATF3 may not effectively protect kidney cells from I/R-induced cell death. The observed outcomes could be explained by system analysis, indicating that ATF3 exhibits context-dependent and opposing effects on pro-apoptotic genes within the p53 pathway [53].

NF-κB is implicated in the pathogenesis of AKI as a master transcription factor of inflammation, triggering the transcription of pro-inflammatory genes [54]. In this study, RDV pretreatment could not decrease the NF‑κB at protein levels compared to the I/R-induced group. In contrast, Yin et al reported that in a mice model of LPS-induced AKI, RDV inhibits the NF‑κB activity effectively, leading to a decline in the inflammasome genes [10]. A possible explanation may reside in the administration route of the drug and the time-dependent pattern of gene expression. We used a single dosage of RDV (25 mg/kg) 1h before the induction of kidney I/R, while Yin et al used the same dosage every 12 h for 7 days. Moreover, they suggest that RDV repressed NF-κB activation during the long term, representing its protective roles in chronic inflammation [10]. It is important to note that ATF3 by direct interaction with p65- NF-κB and epigenetic alterations suppresses the transcription of pro-inflammatory genes [43,44]. In our study, increased levels of ATF3 could not affect p65- NF-κB levels significantly since in our study ATF3 did not significantly increase.

Beyond inflammation and apoptosis, oxidative stress is involved in the pathogenesis of AKI. In this study, RDV pretreatment increased the oxidative stress and decreased the antioxidant capacity of kidney; indicating an increase in free radicals and more kidney damage. It should be noted that the route of RDV administration had significant impacts on the expression levels of the studied proteins. Compared to the intraperitoneal injection, subcutaneous injection of RDV could induce more injury to the kidney; indicated by decreased levels of PGC-1α and antioxidants along with increased levels of oxidant and apoptotic factors.

Although RDV has been shown to impair mitochondrial biogenesis and increase oxidative stress, these effects may not always translate into measurable changes in standard kidney function markers or histological damage within a short time frame. The findings from this study indicate that, under the experimental conditions used, RDV neither exhibited overt nephrotoxicity nor demonstrated protective effects. Likewise, the results of a meta-analysis on 3095 patients indicated that RDV is unlikely to enhance the probability of AKI. Consequently, the AKI incidents reported in patients with COVID-19 receiving RDV may predominantly stem from the SARS-CoV-2 infection itself, pre-existing medical conditions, shock, hypotension, dehydration, and the administration of recognized nephrotoxic agents such as diuretics or nonsteroidal anti-inflammatory drugs [55]. Nevertheless, caution is warranted due to conflicting reports about potential nephrotoxicity associated with its excipient, sulfobutylether-beta-cyclodextrin, accumulation that accumulates in patients with severe kidney dysfunction [12–14].

This study has several limitations. The unilateral renal I/R model with one kidney subjected to ischemia is a reliable and reproducible model for studying acute and chronic kidney injury mechanisms in mice [56]. However, the intact kidney may compensate for the loss of function in the ischemic kidney. Additionally, the early 6-hour time point primarily captures acute injury and molecular pathway activation but may not reveal the full extent of histological alterations or functional recovery versus progression. For future studies, alternative models like the double kidney clip model or unilateral nephrectomy preceding a single vascular clip ischemia may provide complementary insights by avoiding compensatory mechanisms from the contralateral kidney. Moreover, multiple post-reperfusion time points extending to 24–48 hours or beyond are recommended, which will provide a more comprehensive temporal profile of injury, repair, and the intervention’s effects. Studying the effect of remdesivir on the unaffected kidney and other major distant organs is also important to exclude potential off-target effects or systemic toxicity. Further studies are needed to clarify these effects and establish guidelines for safe administration in patients with compromised renal function.

## 5. Conclusions

In summary, the potential mechanisms underlying kidney injury following the administration of RDV include mitochondrial dysfunction and apoptosis of kidney cells. Specifically, decreased mitochondrial biogenesis and increased mitochondrial fission lead to fragmentation of mitochondria, which, in turn, reduces the antioxidative capacity of the kidneys. These factors may exacerbate I/R injury in our kidney model.

## Supporting information

S1 Raw images Western blot raw images. Uncropped western blot gel images for mitochondrial biogenesis and dynamics factors (PGC-1α and Drp-1) in kidney tissues of the studied rats. They marks the section used in Fig 2. Uncropped western blot gel images for p-p53, p-p21, caspase-3, ATF3 in kidney tissues of the studied rats. Mark the sections used in Fig 3. Uncropped western blot gel image for NF-kβ that marks the section used in Fig 4A.
(PDF)

S1 Raw data Raw data of oxidant and antioxidant factors. The raw data of oxidative MDA marker and oxidative factors SOD, TAC, and GPX evaluated in the studied groups. GPX: glutathione peroxidase, MDA: malondialdehyde, SOD: superoxide dismutase, TAC: total antioxidant capacity. Mark the sections used in Fig 4C-4F.
(PDF)
