# Supplementary figures and images for: Remdesivir may exacerbate ischemic acute kidney injury through molecular alterations in PGC-1α and apoptosis pathways: An in vivo study
Source: PLoS One. 2026 Feb 12;21(2):e0336221. doi: 10.1371/journal.pone.0336221 (PMC12900325; doi:10.1371/journal.pone.0336221)

Supporting materials:

Western gel images

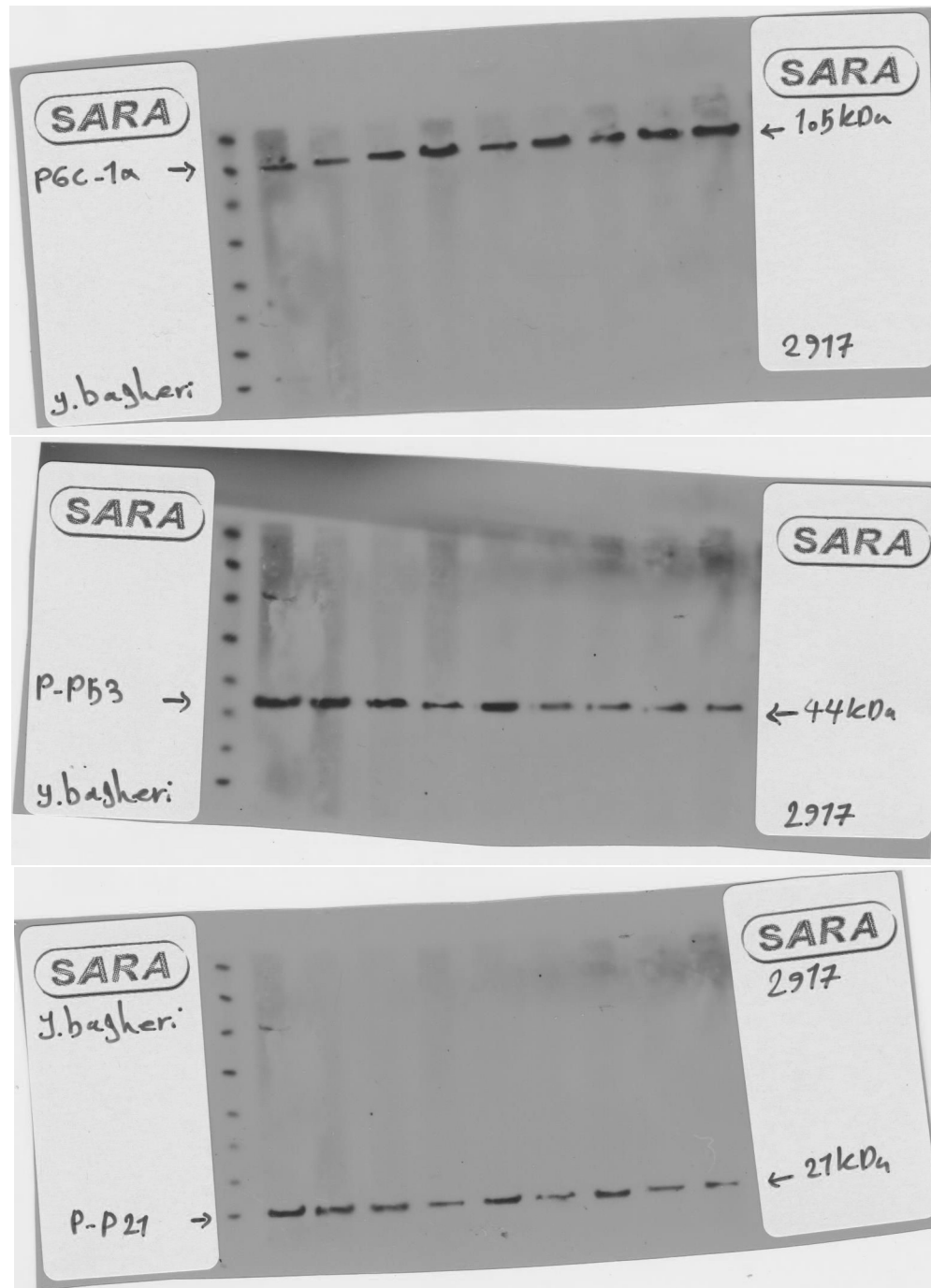

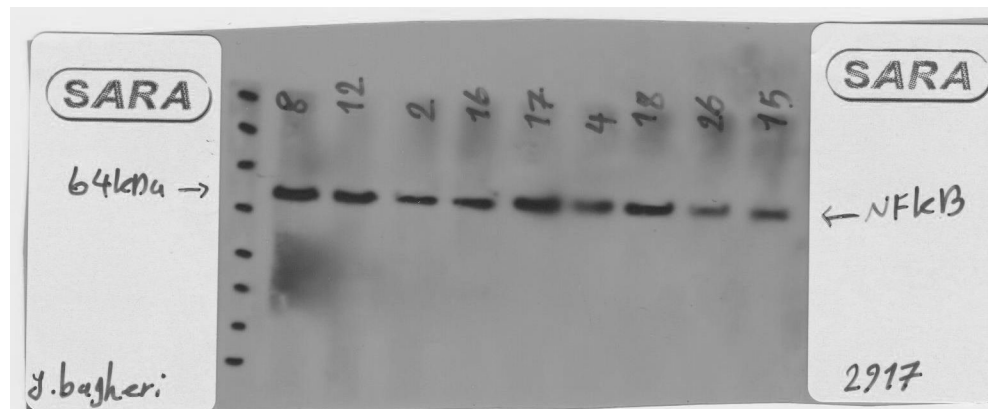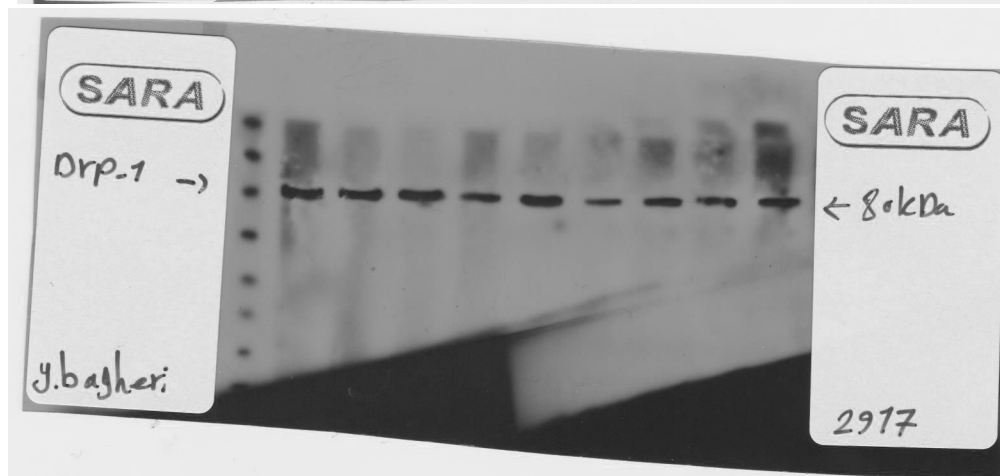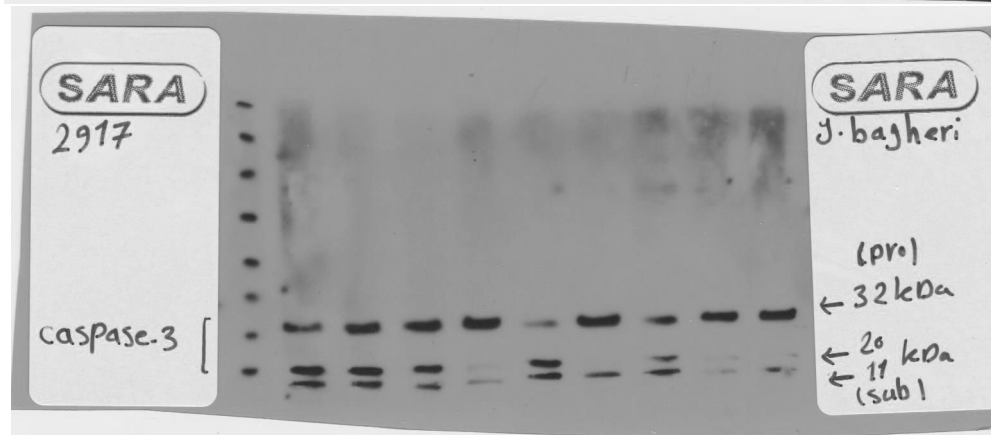

Supplement: S1 Raw images — Uncropped western blot gel images for mitochondrial biogenesis and dynamics factors (PGC-1α and Drp-1) in kidney tissues of the studied rats. They marks the section used in Fig 2. Uncropped western blot gel images for p-p53, p-p21, caspase-3, ATF3 in kidney tissues of the studied rats. Mark the sections used in Fig 3. Uncropped western blot gel image for NF-kβ that marks the section used in Fig 4A. (PDF) [file pone.0336221.s001.pdf]
