# Supplementary material for: Remdesivir may exacerbate ischemic acute kidney injury through molecular alterations in PGC-1α and apoptosis pathways: An in vivo study
Source: PLoS One. 2026 Feb 12;21(2):e0336221. doi: 10.1371/journal.pone.0336221 (PMC12900325; doi:10.1371/journal.pone.0336221)
Supplement: S1 Raw data — The raw data of oxidative MDA marker and oxidative factors SOD, TAC, and GPX evaluated in the studied groups. GPX: glutathione peroxidase, MDA: malondialdehyde, SOD: superoxide dismutase, TAC: total antioxidant capacity. Mark the sections used in Fig 4C-4F. (PDF) [file pone.0336221.s002.pdf]

Sample type	Groups	SOD (U/mg protein)	GPX (U/mg protein)	MDA(nmol/mg protein)	TAC(µmol/ml)
Rat Kidney	Sham	16.1	82.4	1.4	117.1
	Sham	17.8	95	1.4	122.2
	Sham	17.5	87.3	1.3	131.1
	Sham	17.1	89	1.4	112.2
	Sham	19.1	91.3	1.5	103.3
	I/R	15.8	65.2	2.1	84.3
	I/R	13.5	67.1	2.4	98.2
	I/R	11.9	75.3	1.8	86.3
	I/R	14.2	71.2	2.1	92.5
	I/R	12.7	59.6	2.2	102.3
	I/R+Rem+ip	13.3	59.5	2.8	84.3
	I/R+Rem+ip	12.8	60.6	2.3	88.7
	I/R+Rem+ip	12.8	58.6	1.9	93.3
	I/R+Rem+ip	14.2	64.3	2.5	85.3
	I/R+Rem+ip	11.2	50.2	2.2	69.6
	I/R+Rem+sc	9	44.6	3.6	80.8
	I/R+Rem+sc	12.9	37	2.9	63.6
	I/R+Rem+sc	11.1	57.7	2.4	70.1
	I/R+Rem+sc	11.8	40.5	3	71.9
	I/R+Rem+sc	13.8	43.6	3.1	72.9

Injury score	Urea (mg/dl)	Creatinin (mg/dl)	PGC-1α	Nf-kB	Caspase3-Cleaved	Drp-1	p-P21	p-P53	ATF3
0	30	0.8	1	1	1	1	1	1	1
1	34	1	0.75	1.7	2.8	2	2.31	1.2	1.35
1	27	0.5	0.8	1.8	2.31	2.43	2.42	2	1.8
1	35	0.7	0.77	1.75	2.5	2.3	2.34	1.9	1.7
1	28	0.8	0.52	1.56	6.87	2	1.2	1.1	1.35
4	30	0.9	0.68	2	2.88	1.7	2	1.1	1.85
3	47	1.4	0.49	1.3	4.99	1.6	1.2	2	1.1
3	40	1.4	0.39	1.98	9	2.25	2.89	2.5	3
2	43	1.6	0.29	1.35	8.21	2.09	2.69	2.4	2.9
3	38	1.5	0.43	2.6	8.07	2.26	1.3	1.3	1.15
3	46	1.3							
2	52	1.4							
3	52	1.5							
3	50	1.4							
3	42	1.3							
3	55	1.5							
3	53	1.6							
4	48	1.6							
3	53	1.5							
3	58	1.7							
	48	1.3							
	51	1.4							
	57	1.5							
	42	1.8							
